# Effects of Topological Constraints on Penetration Structures of Semi-Flexible Ring Polymers

**DOI:** 10.3390/polym12112659

**Published:** 2020-11-11

**Authors:** Fuchen Guo, Ke Li, Jiaxin Wu, Linli He, Linxi Zhang

**Affiliations:** 1 Department of Physics, Zhejiang University, Hangzhou 310027, China; 11836024@zju.edu.cn (F.G.); 21736027@zju.edu.cn (K.L.); 21936020@zju.edu.cn (J.W.); 2 Department of Physics, Wenzhou University, Wenzhou 325035, China

**Keywords:** molecular dynamics simulations, semi-flexible ring polymers, topological constraints, penetration, chain stiffness

## Abstract

The effects of topological constraints on penetration structures of semi-flexible ring polymers in a melt are investigated using molecular dynamics simulations, considering simultaneously the effects of the chain stiffness. Three topology types of rings are considered: 0_1_-knot (the unknotted), 3_1_-knot and 6_1_-knot ring polymers, respectively. With the improved algorithm to detect and quantify the inter-ring penetration (or inter-ring threading), the degree of ring threading does not increase monotonously with the chain stiffness, existing a peak value at the intermediate stiffness. It indicates that rings interpenetrate most at intermediate stiffness where there is a balance between coil expansion (favoring penetrations) and stiffness (inhibiting penetrations). Meanwhile, the inter-ring penetration would be suppressed with the knot complexity of the rings. The analysis of effective potential between the rings provides a better understanding for this non-monotonous behavior in inter-ring penetration.

## 1. Introduction

A ring polymer is formed by the simple process of joining the ends of a linear polymer chain, corresponding to the so called 0_1_-knot or trivial knot, that is, an unknotted ring polymer. For the semi-flexible rings in the range of the crossover from the rod-like limit to the random coil one, the conformations show a strong anisotropic character relying on the stiffness. As is well known, DNA often exhibits a knotted ring conformation in bacteria and phages [1]. Now, synthetic ring polymers display very complex knotted configurations, such as 3_1_-knot, 4_1_-knot, 5_1_-knot, 6_1_-knot and so forth in Conway’s notation (e.g., *N_k_-*knot denotes the *k*th type of knotted ring with N crossings) [2]. The presence of knots in a ring polymer can be viewed as a self-entanglement phenomenon [3]. The topological constraints of rings has a dramatic effect on the physical properties with respect to their linear counterparts due to the change in the conformational degrees of freedom [4,5]. The most prominent examples are their different scaling behaviors [6,7,8,9,10] and rheological properties [11,12]. First, it has been demonstrated that the size of isolated rings scales with the polymerization degree (*N*) as *D*_g_~*N*^VF^, where *D*_g_ is the diameter of gyration and *V*_F_ ≈ 0.588 is the Flory exponent, respectively. However, the size of linear chain scales as *D*_g_~*N*^0.5^ [7]. Second, no free ends implies that rings do not relax via common reptation which is primarily responsible for linear chains [13]. Unlike the linear chains, the ring polymer melts exhibit self-similar dynamics, thereby yielding a power-law stress relaxation instead of the rubber-like plateau of linear melts [14]. Additionally, the topological constraints dramatically reduces its attainable states in the phase space, resulting in a repulsive effective interaction between two ring chains, while it vanishes between two linear chains or a linear chain and a ring chain [15,16,17].

Absent in systems of linear chains, inter-ring penetration (or inter-ring threading) is a unique feature of ring polymers but the effects of threading is still controversial until now. Earlier studies [18,19] neglect the threading and assume rings to adopt a double-folded annealed branched structure. This so-called annealed tree model [20,21] shows a great agreement with simulations whether on static or dynamic properties so far. However, studies by Michieletto et al. [22,23] and Lee et al. [24,25] believe in the profound effects of the ring threading, responsible for the observed “topologically-driven glassy” behavior. To address this controversy, it is extremely necessary to have a comprehensive understanding of penetration structures of semi-flexible rings. It is quite essential for theoretical simulation and also for experiment of bio-packing [26,27], such as observed in circular DNA of bacteriophage viruses.

In this work, we focus on the penetration structures of three knot types of rings, that is, the 0_1_-knot, the 3_1_-knot and the 6_1_-knot rings [2]. We define the degree of penetration and monitor the internal structure of penetrated rings in detail by changing the complexity of the knot, the bending energy of rings and the number density of system. Meanwhile, we also derive the effective potential between penetrated rings to support our findings about the effects of the topological constraints and chain stiffness on inter-ring penetration. Our results can provide a deeper understanding about the penetration structure of rings, especially for the case of semi-flexible rings.

## 2. Simulation Model and Method

### 2.1. Model

A bead-spring model by Kremer and Grest [28] is used to model the non-concatenated ring polymer, each consisting of *N* spherical monomers with a monomer diameter of *σ* and a mass of *m*. Bonded monomers interact via the finitely extensible nonlinear elastic (FENE) potential [29]

(1) $$U_{FENE}\left(r\right)=-\frac{KR_{0}^{2}}{2}ln\left[1-{\left(\frac{r}{R_{0}}\right)}^{2}\right],$$

where *r* is the distance between two neighboring monomers. *K* = 30*k*_B_*T*/*σ*^2^ is a spring constant and *R_0_* = 1.5*σ* is a finite extensibility to avoid chain crossing, where *k*_B_ is the Boltzmann constant and *T* is the temperature. The chain stiffness is introduced by means of a bending potential between adjacent bonds given by

(2) $$U_{bending}\left(\theta \right)=K_{b}\left(1-cos\left(\theta -\theta_{0}\right)\right),\;$$

where *θ* is the angle between two consecutive bonds with $\theta_{0}=$ (N-2)*π/N and *K_b_* represents the bending energy. For any two monomers, they also interact through a shift and cutoff Lennard-Jones (LJ) potential

(3) $$U_{LJ}\left(r\right)=\{\begin{array}{cc} \;4\epsilon \left[{\left(\frac{\sigma }{r}\right)}^{12}-{\left(\frac{\sigma }{r}\right)}^{6}+\frac{1}{4}\right] & \;\;\;\;\;\;r<r_{C}\\ 0\;\;\;\;\;\;\; & \;\;\;\;\;\;r>r_{C} \end{array},$$

where $r_{c}$ is the cut off distance and fixed at $2^{1/6}\sigma$ and ε is chosen to be $\varepsilon =1k_{B}T$.

### 2.2. Molecule Dynamics Simulation

We perform molecule dynamics (MD) simulations by using the large-scale atomic/molecular massively parallel simulator (LAMMPS) [30]. Reduced units (ε=1, σ=1, m=1 *and τ_0_* = (*mσ*^2^/*k*_B_*T*)^1/2^ = 1 are chosen to be the units of energy, length, mass and time, respectively) and the timestep is *τ* = 0.001$\tau_{0}$. The simulation box size is fixed at 50σ × 50σ × 50*σ* and the periodic boundary conditions are performed in NVT ensemble (canonical ensemble). All ring polymers are placed randomly in our simulation box initially. The corresponding dynamics does not allow for chain crossings to make sure that the topology is preserved. To get a high concentration of rings, we set a scaling factor big enough. Thousands of steps are used to obtain the local equilibrated conformation after each step of scaling, making sure that the scaling does not affect the topology of rings. After that, an additional equilibration run (up to 9 × 10^8^ steps) is performed to get the steady penetration structures of rings.

**Figure 1:**
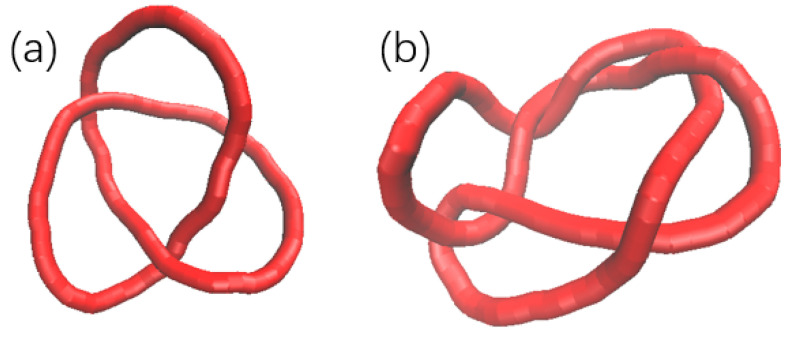
Typical snapshots for two topological knots (**a**) 3_1_-knot and (**b**) 6_1_-knot.

In our simulation, three topology types or knotted rings are considered: 0_1_-knot (the unknotted or trivial knot), 3_1_-knot (the trefoil knot) and 6_1_-knot ring polymers, named in Conway’s notation [2]. The typical snapshots for latter two knots are shown in Figure 1. Here the bending energy *K*_b_ ranges from *K*_b_ = 0 to *K*_b_ = 100 in the units of *ε,* corresponding to flexible, semi-flexible and stiff ring polymers, respectively. Intermediate *K*_b_ values correspond the semi-flexible rings. The number density is defined as *ρ* = M*N/L^3^, where *M* is the number of rings and *N* is the number of monomers per ring. Firstly, *ρ* = 0.1 with *N*=128 is considered, that is, *M* *=* 98. Then the number density *ρ* varies in the range of 0.05~0.4 with *N* = 128, that is, *M=* 49~392. 

The effective potential of mean force (PMF) between a pair of rings can be calculated directly from NVT simulations given by References [31,32,33,34]

(4) $$\beta V_{eff}\left(r\right)=-\ln\rho^{\prime }\left(r\right),$$

where $\beta ={\left(k_{B}T\right)}^{-1}$ and $\rho^{\prime }\left(r\right)$ is the probability distribution of the centers-of-mass of rings. Since the range of interaction is finite (of the order of $D_{g}$, the average diameter of gyration), we do not need to calculate those distances which are longer than $D_{g}$. While for the short distance ($r<D_{g}$), due to the topology repulsion, there are large energy barriers which prevent an effective exploration of the configurational space within the available computer time. To overcome this, we use the umbrella sampling [35,36] to calculate $\rho^{\prime }\left(r\right)$ where the biased potential is a simple harmonic spring

(5) $$V_{bias}\left(r\right)=\frac{k_{j}}{2}{\left(r-r_{j}\right)}^{2},$$

where $k_{j}=4.0\varepsilon /\sigma^{2}$ and $r_{j}$ stands for the free length of the spring at different windows. We perform the simulations starting from $r_{j}=0$ and increase it up to a maximum value which is much larger than $D_{g}$ for different rings to get the biased distributions ${\rho^{\prime }}_{j}^{\left(b\right)}\left(r\right)$. To eliminate the influence of the harmonic potential on total probability distribution $\rho^{\prime }\left(r\right)$, we have to employ the weighted histogram analysis method (WHAM) [33,34,35,36,37] to combine the biased distributions ${\rho^{\prime }}_{j}^{\left(b\right)}\left(r\right)$ at each window. In order to achieve that, we need derive $\rho^{\prime }\left(r\right)$ in terms of unbiased densities ${\rho^{\prime }}_{j}^{\left(u\right)}\left(r\right)$ at each simulation window [34,35]

(6) $$\rho^{\prime }\left(r\right)=\sum_{j=1}^{N^{\prime }}p_{j}\left(r\right){\rho^{\prime }}_{j}^{\left(u\right)}\left(r\right),$$

with

(7) $${\rho^{\prime }}_{j}^{\left(u\right)}\left(r\right)=e^{\beta \left(V_{j}\left(r\right)-f_{j}\right)}{\rho^{\prime }}_{j}^{\left(b\right)}\left(r\right),$$

where $V_{j}$ is the biased potential and $f_{j}$ is free energy parameter arising from the addition of the biased potential. The normalization condition requires the sum of $p_{j}\left(r\right)$ is equal to 1 and they are chosen so as to minimize the statistical error. Then we can get

(8) $$\rho^{\prime }\left(r\right)=\sum_{j=1}^{N^{\prime }}c_{j}\left(r\right){\rho^{\prime }}_{j}^{\left(b\right)}\left(r\right),$$

with coefficient

(9) $$c_{j}\left(r\right)=1/\sum_{j^{\prime }}^{N^{\prime }}e^{-\beta \left(V_{j^{\prime }}\left(r\right)-f_{j^{\prime }}\right)}.$$

Up to now, we already get the total probability distribution $\rho^{\prime }\left(r\right)$ except that $f_{j}$ are unknown. In fact, $f_{j}$ can be obtained self-consistently by

(10) $$e^{-f_{k}}=\sum_{i=1}^{N^{\prime }}\sum_{j=1}^{n_{i}}\frac{e^{-\beta V_{k}\left(r_{i,l}\right)}}{\sum_{j=1}^{N^{\prime }}n_{j}e^{-\beta \left(V_{j}\left(r_{i,l}\right)-f_{j}\right)}},$$

where $n_{j}$ is the number of sampled conformations at *j*th window. $r_{i,l}$ is the reaction coordinate value for conformation snap *l* in the simulation window *i*.

### 2.3. KMT Algorithm

**Figure 2:**
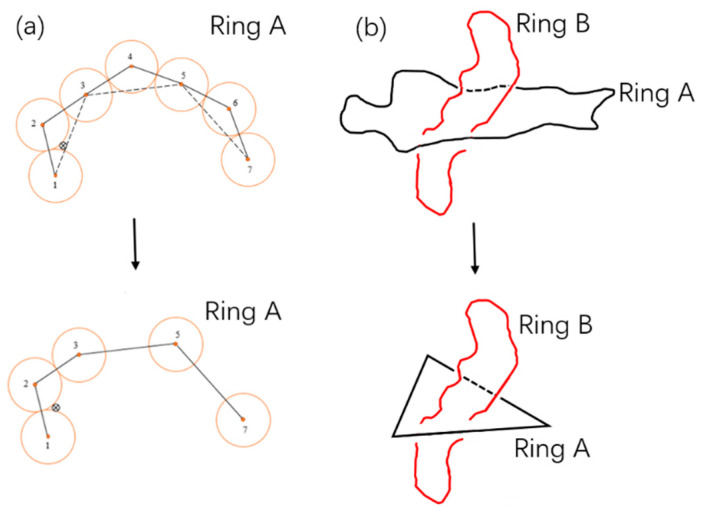
(**a**) Illustration of the KMT algorithm [38,39,40]. Ring A is passively threaded by ring B, which is omitted for simplicity with only one dot marking a cross bond. Monomers 1~7 are part of the ring A. After the first reduction, monomers 4 and 6 are removed as triangle 345 and 567 are not intersected by Ring B. (**b**) If the ring A is threaded by ring B, ring A can be reduced finally into a triangle after many KMT reductions.

To define whether a chain is penetrated by others, that is, a definition of threading, we introduce the improved KMT algorithm firstly suggested by Koniaris and Muthukumar [38], developed independently by Taylor [39] and referred as KTM reduction by Virnau [40]. As shown in Figure 2, assuming that the ring A is penetrated by another ring B. The KMT algorithm divides the ring A into a series of consecutive triangles of adjacent monomers, such as 123, 345 and 567. The cross denotes the intersection point of which ring A is penetrated by ring B in the case of reduction shown in Figure 2. After the first reduction, monomers 4 and 6 are removed since neither triangle 345 nor 567 is intersected by any part of ring B, while monomer 5 is retained. Through enough iterations of reduction, ring A is reduced into a simple triangle 123. The surface of the ring A is composed of triangle 123, while other triangles are removed at each reduction (e.g., 345 and 567 at the first reduction). Among all the possible surfaces by different routines of reduction, we can find the minimal surface [25,41] and the corresponding final triangle (e.g., the case of Figure 2b) to define the threading. With the definition of threading produced by a pair of rings, we call them one passive ring (A) and one active ring (B) respectively. The ring A is passively threaded by ring B, which is actively threading the ring A [21]. In the following, all of the average statistics goes through all rings.

## 3. Results and Discussion

**Figure 3:**
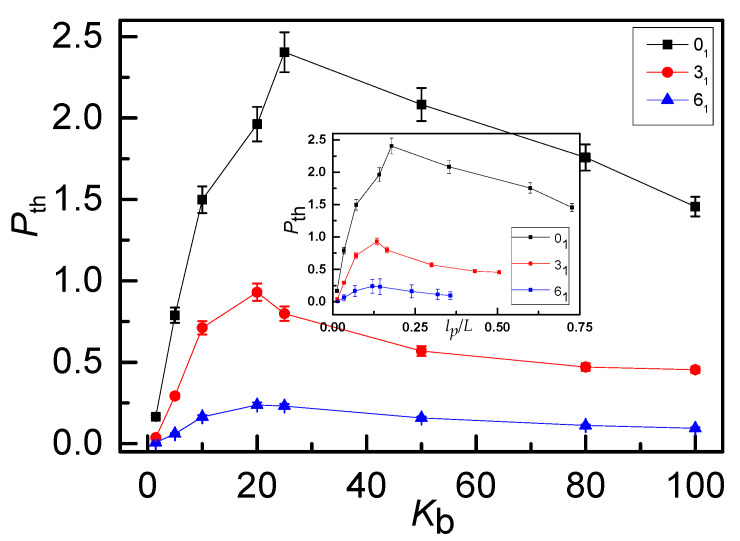
Average threading number *P_th_* shows a non-monotonous dependence on bending energy *K_b_* for 0_1_-knot, 3_1_-knot and 6_1_-knot ring polymers. The peak appears at the intermediate stiffness corresponding to the semi-flexible case. Inset: Average threading number *P_th_* as a function of *l_p_/L*, where *l_p_* denotes the persistence length and L is the contour length of the chain.

We firstly focus on the degree of ring threading with different knot types and chain stiffness. An average threading number *P*_th_ is defined as the number of rings penetrated by a single chain. Here three knot types are considered: 0_1_-knot, 3_1_-knot and 6_1_-knot rings respectively. The *P*_th_ varied by bending energy *K*_b_ for three knot types is displayed in Figure 3. It can be seen that *P*_th_ drops rapidly from 0_1_-knot to 3_1_-knot and even to 6_1_-knot rings, due to the decrease of the free space that can be penetrated by other rings due to the internal topology. For the given ring topology structure, for example, 0_1_-knot ring, as *K*_b_ increases, *P*_th_ increases rapidly first and then decreases slightly, exhibiting a maximum value *P*_th-max_ corresponding to an intermediate stiffness. A similar *K*_b_ dependence of *P*_th_ is presented for other two knots. For flexible rings, the knot has little influence on *P*_th_ as rings shrink into crumpled globular conformations, highly unfavorable for penetration. With the increase of bending energy *K*_b_, rings can expand and adopt open-up configurations that facilitate inter-ring penetration, in great agreement with the results by Bernabei et.al [42]. However, the intrinsic mechanism on this non-monotonous dependence on bending energy *K*_b_ should be further explored in the following. As well known, the persistence length *l_p_* is an important parameter to describe the conformation of semiflexible polymer. The ratio of *l_p_/L* depends on the bending energy (*K*_b_), chain length (*N*) and the chain topological structure is given in Figure S1 (see Figure S1 in Supporting Information).

**Figure 4:**
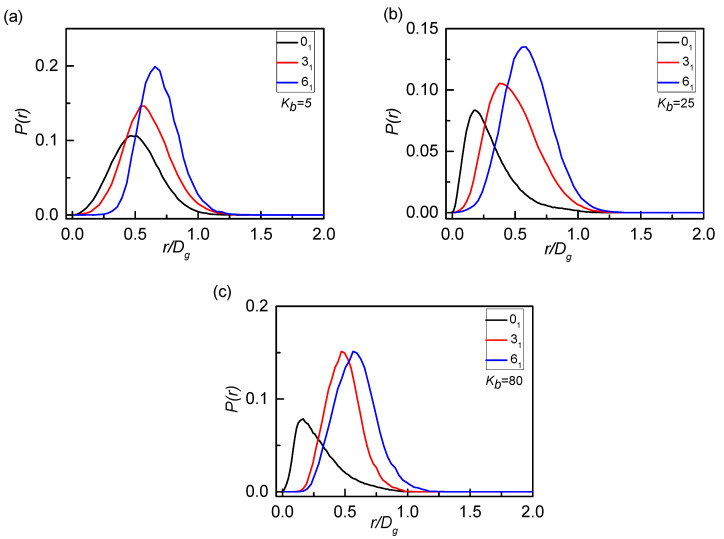
Probability density distributions *P*(*r*) of penetrated rings for different bending energies *K*_b_ corresponding to the case of (**a**) flexible rings, (**b**)semi-flexible rings, and (**c**) rigid rings with three knot types. *P*(*r*) denotes the probability distribution for the centers of mass between two penetrated rings. The r is rescaled by *D_g_* thus the normalization condition satisfies $\int^{\;}P\left(r\right)dr=$ *D_g_*.

In detail, we turn to detect the internal structure of penetrated rings to illustrate the expansion of the rings. The probability density distributions *P*(r) of the centers of mass between two penetrated rings for three knot types are shown in Figure 4. The size of the rings is changed with the topology thus we rescale the distance r by *D_g_*, which is more comparable to discuss about the threading structures. For flexible rings (*K*_b_ = 5) shown in Figure 4a, the peak appears at a far distance range of r = 0.5~0.75$D_{g}$, which means a weak penetration depth owing to the shrinkage of flexible rings. For semi-flexible rings (*K*_b_ = 25) and stiff rings (*K*_b_ = 80) shown in Figure 4b,c, the peaks shift to a short distance, especially for the 0_1_-knot rings (r^*^ ≈ 0.20 for *K*_b_ = 25). The results also confirm the fact the chain stiffness can help rings swell and free up more space for other rings, favorable for inter-rings penetration. For different knot types, the more complex the topological constraints, the farther the peak position and the lower the penetration depth, which is quite consistence with the results of Figure 3.

**Figure 5:**
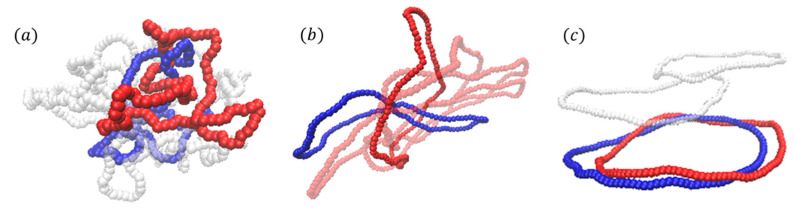
Illustration of typical threading structures of 0_1_-knot rings with different bending energies. (**a**) *K*_b_ = 5, flexible rings adopt coiled conformation. (**b**) *K*_b_ = 25, the semi-flexible rings pierce others forming a deep penetration. (**c**) *K*_b_ = 80, rigid rings get parallel with each other resulting in a slight threading.

For a better view, the typical penetration structures of 0_1_-knot rings with different bending energies K_b_ are investigated in Figure 5. The blue ring presents the active ring B and the red is the passive one A, while the white ring is not penetrated by the blue ring. Flexible rings barely penetrate each other due to the crumpled and filled structures shown in Figure 5a. For semi-flexible rings in Figure 5b, it is the deepest penetration between rings, which are involved in several rings. The further increase of bending energy *K*_b_ leads to an improve of the flatness of the polymer contours in Figure 5c. The planar rigid rings tend to be arranged parallel to each other, thus weakening the penetration depth with respect to their semi-flexible counterparts, showing a strong anisotropic character, which is strengthened by the increase of the stiffness.

**Figure 6:**
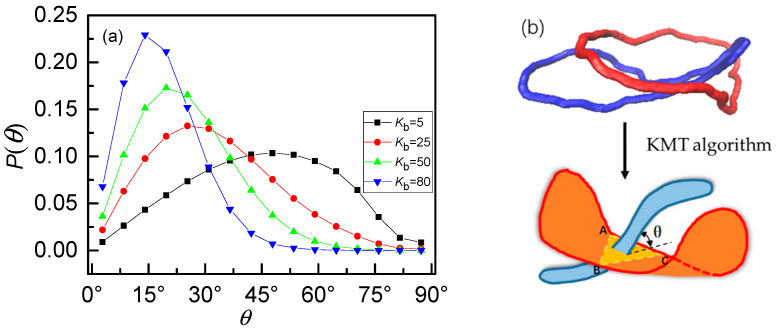
(**a**) Probability distributions *P*(*θ*) of threading angle for 0_1_-knot rings with different bending energies *K*_b_ shows an obvious transition from isotropic character to anisotropic character. (**b**) The upper panel shows the typical snapshot for two penetrated rings and the lower panel is the simplified case to show the definition of the threading angle as a triangle intersected a line based on the KMT algorithm.

As discussed above, the effects of increasing stiffness have two aspects. One is the expansion of the internal space of rings. The other is the anisotropic character which seems to weaken the penetration. We will focus on the anisotropic character in the following. Our finding shows that the competition of these two effects results in the non-monotonous dependence of *P_th_* on stiffness. Only focus on the case of 0_1_-knot rings, the probability distributions *P*(*θ*) of threading angle for different bending energies *K*_b_ are shown in Figure 6a. As we have mentioned in the KMT algorithm, one ring can be reduced into a triangle penetrated by another ring satisfying the minimal surface principle. Therefore, the threading angle *θ* is defined as the angle between a line and a triangular plane (ABC) based on the KMT algorithm, as shown in the Figure 6b. For the flexible rings, the black line exhibits an approximate Gaussian distribution with a mean angle of ~45°. It indicates the conformation of flexible rings is isotropic like a collapsed ball, as displayed in Figure 5a, leading to a full angle distribution. Flexible rings penetrate each other slightly due to the strong intramolecular barriers [42,43,44]. With the increase of bending energy *K*_b_, not only the peak value of *P*(*θ*) increases but also the position of the peak shifts to a smaller angle. As well known, the shapes of rings change from prolate, crumpled structures to planar, rigid rings caused by the increase of chain stiffness, which can dramatically destroy the isotropic of the rings. Rigid rings (*K*_b_ = 80) tend to be parallel with each other as a result of the topology potential [42,43,44], leading to a quite small threading angle and even close to 0°. In a word, there is a typical transition from isotropic character to anisotropic character as chain stiffness increases.

**Figure 7:**
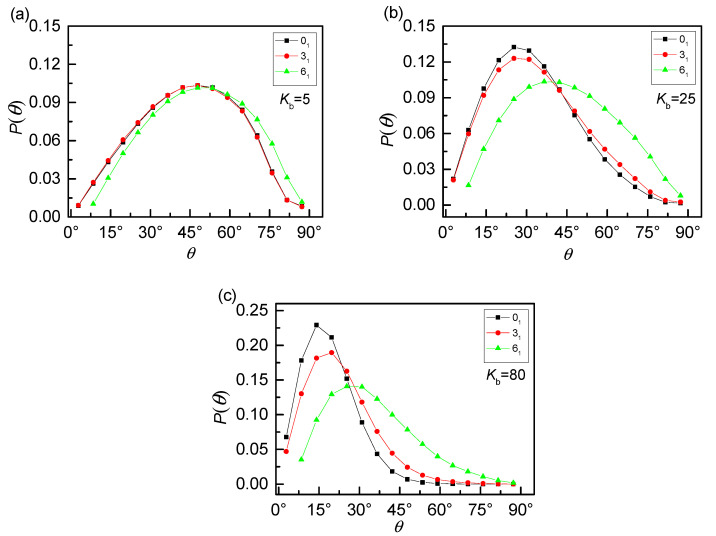
Probability distributions *P(θ)* of threading angle for different bending energies (**a**) flexible rings, (**b**) semi-flexible rings, and (**c**) rigid rings with three knot types. The topology constraints suppress the anisotropic character, especially at the case of rigid rings.

Corresponding to the three knot types, the probability distributions *P*(*θ*) of threading angle for different bending energies are shown in Figure 7. For the flexible case, there is little difference among 0_1_-knot, 3_1_-knot and 6_1_-knot rings, because all of them interact as an isotropic ball, corresponding to a full angle distribution. With the increase of bending energy *K*_b_, the difference among the three knot types is highlighted, especially for rigid rings (*K*_b_ = 80) shown in Figure 7c. The peaks of *P*(*θ*) are suppressed and shifted to a broad angle as the knot complexity of the rings increases, which actually strengthens the isotropic of rings. As a result, the more rigid of the rings, the stronger of the topology constraints.

**Figure 8:**
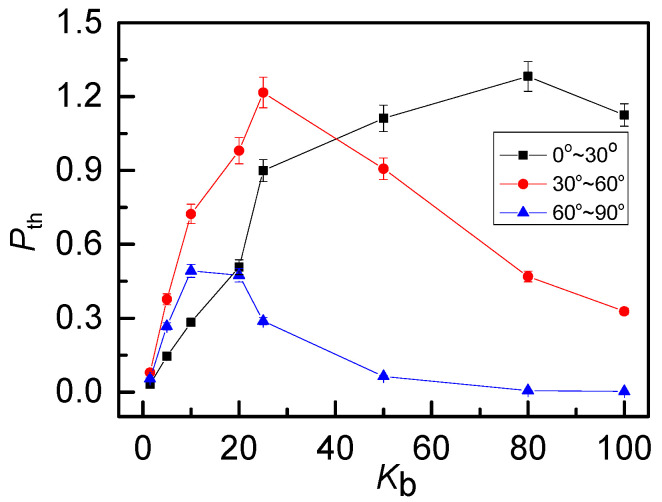
Average threading number *P*_th_ of 0_1_-knot rings within different angel ranges as a function of bending energy *K*_b_ shows a strong anisotropic character. For rigid rings, the penetration of medium and big angles are suppressed due to the parallel structures.

The probability distributions of *P*(θ) indicate that anisotropic plays an important role on the penetration structure. To explain the effect of anisotropic character on threading degree, we subdivide the threading angle *θ* (0°~90°) into three angle ranges: small angle (0°~30°), medium angle (30°~60°) and large angles (60°~90°). Corresponding to the black line in Figure 3, the threading level *P*_th_ of 0_1_-knot rings vs bending energy *K*_b_ for three angle ranges are plotted separately in Figure 8. In general, the proportion of small angle threading (black line) increases monotonously along with *K*_b_. It indicates that the increase of chain stiffness strengthens the anisotropic penetration. Therefore, the stronger the rigidity, the more penetration of the small angle will be. For medium angles (red line) and large angles (blue line), as *K*_b_ increases, *P*_th_ both increase first because of the expansion of the free space, then decrease dramatically owing to the anisotropic character. Especially for the case of large angles (60°~90°), the proportion of penetration dropped sharply or even close to 0, which fits well with the fact that rigid rings tend to be parallel with each other.

**Figure 9:**
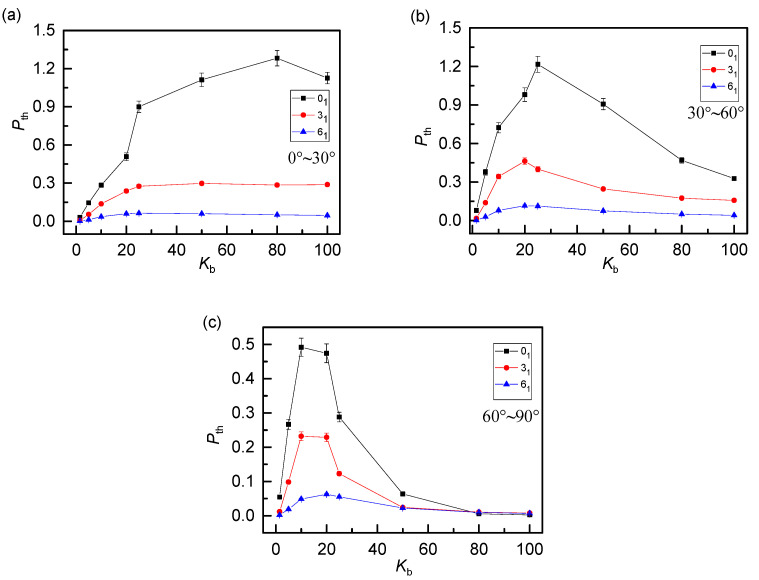
Average threading number *P*_th_ within angles of 0°~30° (**a**), 30°~60° (**b**) and 60°~90° (**c**) as a function of bending energy *K*_b_ for three knot types. The anisotropic character and expansion effects are both suppressed by the complex topology.

We look into the influence of topological constraints on different threading angles as well. As shown in Figure 9, the threading level of 0_1_-knot, 3_1_-knot and 6_1_-knot rings present a similar dependence on *K*_b_ for three angle ranges. In detail, the more complex the topological constraints, the smaller the change in threading level *P*_th_, owing to the decrease of the free space for penetration. What’s more, the complex topology also weakens the anisotropic character, resulting in the domain of the medium threading angles, which is also obvious in Figure 7 as well. Look closely at Figure 9a, there is a little decrease for 0_1_-knot rings at a high bending energy (after *K*_b_ = 80). Rigid rings penetrate others nearly in parallel, where a ring can penetrate only a small amount of other rings, as visually presented in Figure 5c. This is responsible for a slight decline in threading level corresponding to the small threading angle.

**Figure 10:**
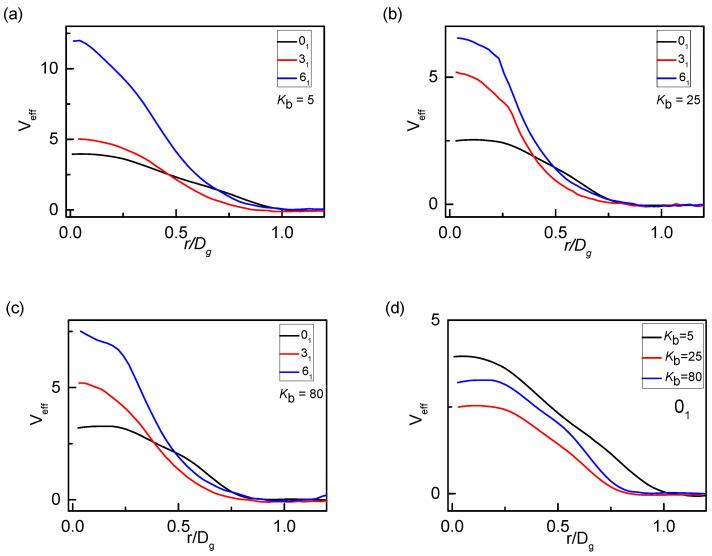
Potential of mean force (PMF) of three knot types (**a**–**c**) and 0_1_-knot rings shows the strong repulsion due to the topology constraints at short distances, resulting in the shift of the peak of *P(r)* in Figure 3 (**d**) PMF for different bending energies *K_b_* present a similar non-monotonous dependence on *K*_b_.

The effective potential of mean force (PMF) between a pair of rings *V*_eff_ for three knot types [45], obtained by umbrella sampling MD, is calculated in Figure 10. For all cases, *V*_eff_ has decayed to zero as r ≈ *D*_g_. From Figure 10a–c, it can be seen that the amplitude of the effective potential at short distances increases with the complexity of the knot, that is, the effective potential of 0_1_-knot rings is lower than 3_1_-knot or 6_1_-knot rings. Combined with the results of Figure 4, take *K*_b_ = 25 for example, the threading peaks of 0_1_-knot rings approximately occur at a distance of 0.2*D*_g_, while 0.4*D*_g_ and 0.6*D*_g_ for 3_1_-knot rings and for 6_1_-knot rings, respectively. Corresponding to the PMF in Figure 10b, at r = 0.2*D*_g_, the potential of 0_1_-knot rings is much lower, while at r = 0.4*D*_g_ or r = 0.6*D*_g_, 3_1_-knot and 6_1_-knot rings are lower than 0_1_-knot rings. It also confirms that 0_1_-knot rings penetrate with each other at a much shorter distance, while rings with complex knots occur at a longer distance. Correspondingly, the effective interaction preferentially occurs at a short distance providing more free space available for inter-ring penetration and causing a deep penetration. Therefore, the threading level of 0_1_-knot rings is much higher than rings with complex knots, shown in Figure 3. Additionally, the PMF of only 0_1_-knot rings for different bending energies *K*_b_ is presented in Figure 10d. The effective potential decreases first and then increases with the increase of bending energy, exactly explaining non-monotonous dependence of the threading level *P*_th_ on chain stiffness. Narros, et. al., also proposed that the penetration conformation for 0_1_-knot rings is quite common for r < 0.25*D*_g_, resulting in the cost in free energy which can be approximately estimated by a Flory theory [3].

**Figure 11:**
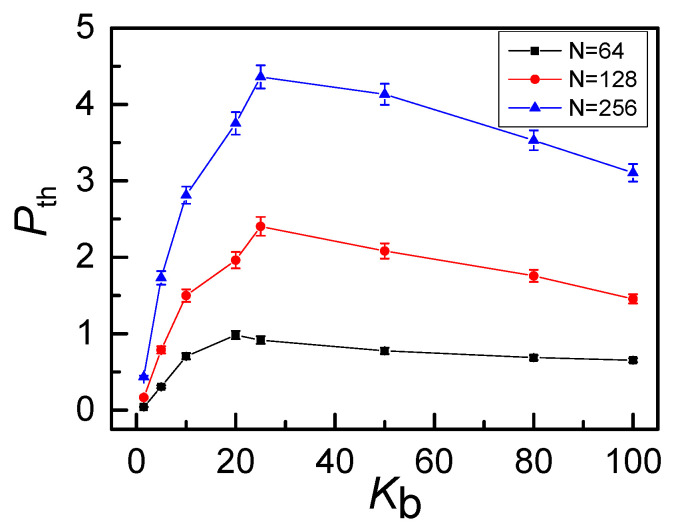
Average threading number *P_th_* increases with the chain length (*N*) and has a peak at *K_b_* = 20~25 for 0_1_-knot ring polymers at *ρ* = 0.1.

**Figure 12:**
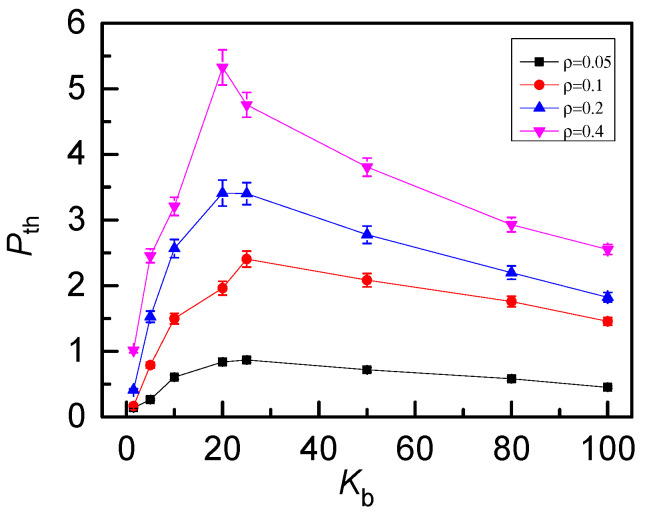
Average threading number *P*_th_ of 0_1_-knot rings as a function of bending energy *K*_b_ for different number densities *ρ*. *P*_th_ increases with the increase of the number density.

**Figure 13:**
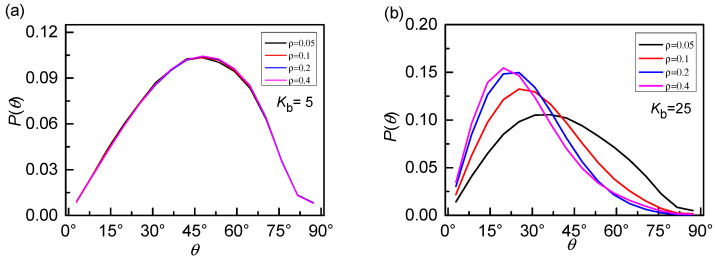

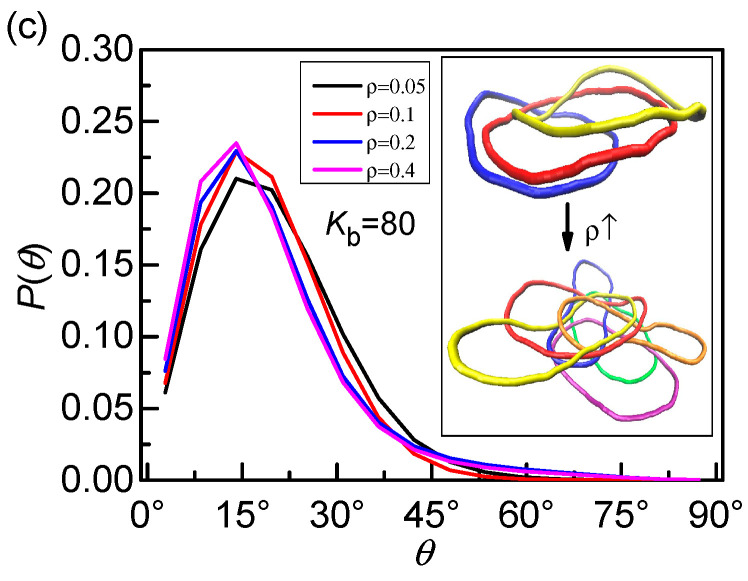
Probability distributions *P*(*θ*) of threading angle for different bending energies *K*_b_ corresponding to the case of (**a**) flexible rings, (**b**)semi-flexible rings, and (**c**) rigid rings with different number densities *ρ*. The anisotropic character is strengthened by the increase of the density at the intermediate stiffness due to the excluded volume effect. The inset in (**c**) shows the rigid rings tend to be arranged parallel to each other with the increasing of number density *ρ.*

Figure 11. The peak position shifts to the small bending energy a little when chain length increases from *N* = 64 to *N* = 256. For example, the peak is located at *K_b_* = 25 for *N* = 256 and at *K_b_* = 20 for *N* = 128 or 64. Meanwhile, the longer of the ring, the larger of the free space for penetration resulting in an increase of *P_th_* for longer rings. Average threading number P_th_ decreases with knot topology and has a peak at *K*_b_ = 20~25 for a fixed length *N* = 256 is given in Figure S2 (see Figure S2 in Supporting Information). As for the density, we focus on the case of 0_1_-knot rings and *ρ* = M*N/L^3^, ranging from 0.05 to 0.4 by changing the number of rings *M.* The threading level *P*_th_ vs bending energy *K*_b_ for different number densities *ρ* are shown in Figure 12. The non-monotonous behavior also becomes more and more prominent with increasing number density. Meanwhile, as *ρ* increases, the amplitude *P*_th_ increases greatly, since more rings have more opportunities to contact, which is available for inter-ring penetration. Then, the effects of number density *ρ* on the probability distribution *P*(*θ*) of threading angles is also presented in Figure 13. The number density has little effect on flexible rings due to the isotropic shrinkage shown in Figure 13a. The anisotropic character of rings is highlighted by the increase of chain stiffness. For semi-flexible rings (*K*_b_ = 25) shown in Figure 13b, the peak of *P*(*θ*) slightly shifts to a smaller angle with the increasing of the number density *ρ*, resulting from the fact that the anisotropic penetration is strengthened by the excluded volume effect for a large number density *ρ*. For rigid rings (*K*_b_ = 80) shown in Figure 13c, the chain stiffness absolutely dominates the anisotropic penetration, unaffected by number density. The corresponding typical structures of rigid rings are displayed in the inset. The planar rigid rings tend to be arranged parallel to each other with a quite small threading angle of ~15°.

## 4. Conclusions

The penetration structures of the semi-flexible ring polymers have been studied by MD simulations. With the implementation of the improved KMT algorithm, the degree of penetration and the internal structures have been characterized by the average threading number *P*_th_ and probability distribution *P*(*r*) of penetrated rings, respectively. Our results show that the penetration degree of rings does not increase monotonously with the chain stiffness. It depends on the balance between coil expansion (favoring penetrations) and stiffness (inhibiting penetrations). As the increase of chain stiffness, the expansion of ring conformations results in more space for penetration, while a subsequent decline in penetration results from the anisotropic character of semi-flexible rings. The anisotropic character of semi-flexible rings, which can be characterized by the probability distribution *P*(*θ*) of threading angle and corresponding average threading number *P*_th_ for different angle ranges, is further confirmed not favoring the penetration. The analysis of effective potential between a pair of rings provides a better understanding for this non-monotonous behavior in inter-ring penetration. Furthermore, it is shown that the penetration would be suppressed with the knot complexity of the rings. Our results reveal the essential roles of topological constraints and chain stiffness on inter-ring penetration.

## Supplementary Materials

The following are available online at https://www.mdpi.com/2073-4360/12/11/2659/s1.
