# Supplementary material for: Effects of Topological Constraints on Penetration Structures of Semi-Flexible Ring Polymers
Source: Polymers (Basel). 2020 Nov 11;12(11):2659. doi: 10.3390/polym12112659 (PMC7696204; doi:10.3390/polym12112659)
Supplement: Supplementary file 1 [file polymers-12-02659-s001.pdf]

Supporting information

Effects of topological constraints on penetration structures of semi-flexible ring polymers

Fuchen Guo [1], Ke Li [1], Jiaxin Wu [1], LinLi He [2], and Linxi Zhang [1]

[1] Department of Physics, Zhejiang University, Hangzhou 310027, Zhejiang, China

[2] Department of Physics, Wenzhou University, Wenzhou 325035, Zhejiang, China

S1 Persistence length (l_p) of knotted ring polymers

Persistence length is an important parameter to describe the conformation of semiflexible polymer. There are four means to define the persistence length.[1-3]

(1) Persistence length can be obtained from the average projection of the end-to-end vector on the first bond of the chain $\vec{l_1}$ for infinitely long chains

$$l_p = \langle \sum_{i=1}^{N-1} \vec{l_i} \vec{l_1} \rangle / \langle l \rangle. \quad (1)$$

(2) It also can be given by the average cosine of the bond angle in a chain

$$l_p = \langle l \rangle / (1 - \langle cos\theta \rangle). \quad (2)$$

Where θ is a complementary angle to a valence angle.

(3) It also can be determined from the exponential decay of orientation correlation in a chain

$$\langle \vec{u}(r) \bullet \vec{u}(0) \rangle = \exp(-r/l_p). \quad (3)$$

(4) It is also computed from the formula derived for the worm-like chain model (WLC) relating the mean-square end-to-end distance of a chain $<R^2>$ to its persistence length

$$\langle R^2 \rangle = 2 l_p \mathrm{L} - 2 {l_p}^2 (1 - \exp(-\mathrm{L}/l_p)), \quad (4)$$

where L is the contour length of the chain, and $<R^2>$ is the mean-square end-to-end distance of a chain. Due to the special topology structure of knotted semiflexible ring polymers, it is difficult to calculate the persistence length of semiflexible rings according to the definitions (1) and (4). We calculated the persistence length according to the definition (3) and the results are given in Figure S1. The ratio of l_p/L depends on the bending energy (K_b), chain length (N) and the chain topological structure, see Figure S1. The ratio of l_p/L increases with bending energy (Kb) for different knot topology or different chain length. Ring polymers with the complicated topological structure have a small persistence length. For example, $l_p/L=0.726$ for 0_1-knot rings and decreases to $l_p/L=0.357$ for 6_1-knot rings at $K_b=100$, see Figure S1(a). Meanwhile, l_p/L decreases when the chain length increases, see Figure S1(b). For example, $l_p/L=1.01$ decrease to $l_p/L=0.365$ when the chain length increases from N=64 to 256 for 0_1-knot ring polymers with $K_b=100$.

(a)

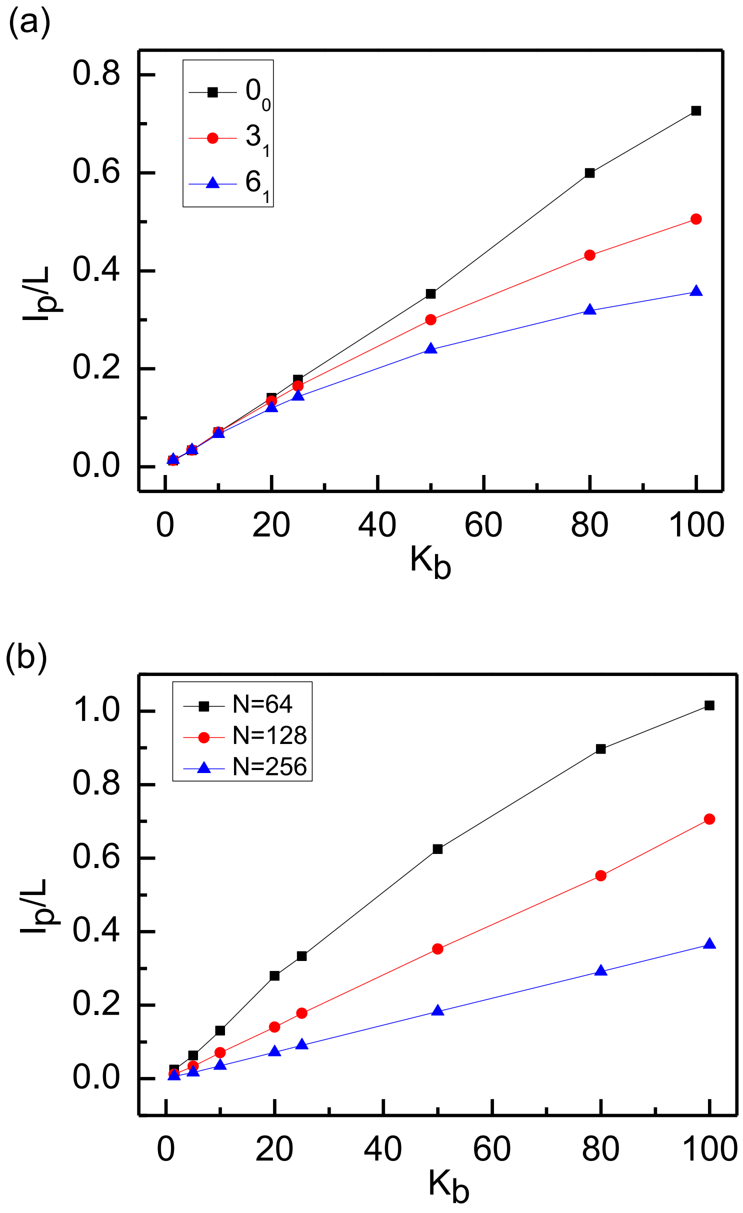

Figure S1: (a) The ratio of l_p/L increases with bending energy K_b as well as decrease with knot topology of ring polymers for N=128 at ρ=0.1. (b) The ratio of l_p/L increases with bending energy K_b as well as decreases with chain length for 0_1-knot ring polymers at ρ=0.1. Here l_p is the persistent length of the chain and L is the contour length of the chain.

S2 Effects of longer chain length (N) on average threading number (P_{th}) of ring polymers

Figure S2 shows average threading number P_{th} as a function of bending energy (K_b) with different knot topology (0_1-knot, 3_1-knot and 6_1-knot) for a longer ring. Average threading number P_{th} decreases with knot topology and has a peak at K_b=20~25 for a fixed length N=256 and a fixed number density ρ=0.1. The penetration structures rely really on the knot topology and chain length of ring polymers. The dependence of P_{th} on the rigidity is same as the case of N=128 shown in the paper.

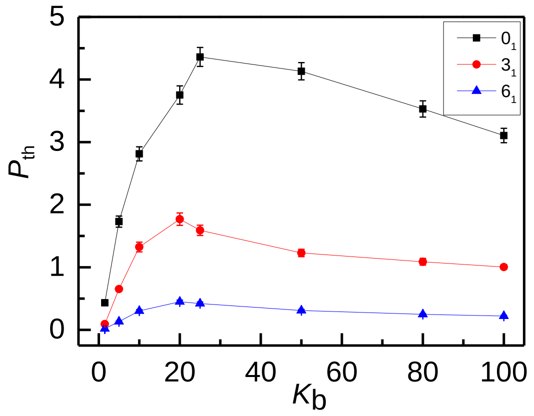

Figure S2. Average threading number P_{th} decreases with knot topology and has a peak at K_b=20~25 for a fixed length N=256 and a fixed number density ρ=0.1.

References:

1. Cifra, P. Differences and limits in estimates of persistence length for semi-flexible macromolecules, ***Polymer***, 2004, 45, 5995-6002.
2. Cifra, P.; Benkova, Z.; Bleha, T. Persistence lengths and structure factors of wormlike polymers under confinement, ***J. Phys. Chem. B***, 2008, 112, 1367-1375.
3. Zhang, J. Z.; Peng, X. Y.; Liu, S.; Jiang, B.P.; Ji, S.C.; Shen, X. C. The persistence length of semiflexible polymers in lattice Monte Carlo simulations, ***Polymers,*** 2019, 11, 295
